# Suicide in Iran: The Facts and the Figures from Nationwide Reports

**Published:** 2017-01

**Authors:** Hossein Hassanian-Moghaddam, Nasim Zamani

**Affiliations:** 1 Toxicological Research Center, Department of Clinical Toxicology, Loghman-Hakim Hospital, Shahid Beheshti University of Medical Sciences, Tehran, Iran.; 2 Excellence Center of Clinical Toxicology, Ministry of Health and Medical Education, Tehran, Iran.

**Keywords:** *Death*, *Epidemiology*, *Iran*, *Suicide*, *Violence*

## Abstract

**Objective: **Data on national rates of suicide is limited in Iran, preventing an action plan for health scope of suicide prevention. The current brief study was conducted to review available national published data on suicide and to interpret the probable discrepancies.

**Method:** We evaluated all 20-year recent published original articles on committed suicides searching Iranian scientific databases, PubMed, and Google Scholar with the keywords of suicide, mortality, and Iran.

**Results:** Articles showed an overall increased trend of suicidal deaths in Iran. Discrepancies existed regarding suicide rate and demographic characteristics among 9 English and Persian published articles. Although a suicide rate of 6.2 per 100 000 was reported in 2003, almost 31 times greater than 1991, an average suicide rate of 9.9 per 100 000 was calculated based on data interpretations.

**Conclusion**: Apparently, Iran has had the highest increase in suicide-related deaths among Eastern Mediterranean Region (EMR) and Islamic countries during the recent decades. National policies to prevent suicide have not been efficient enough, and urgent intervention is needed.‏

There are few publications concerning national suicide data in Islamic republic of Iran. Some discrepancies were detected, while reviewing the existing articles regarding their presentation and interpretation.

The present study aimed at reviewing the available national published data on suicide in Iran, obtaining a view of prevalence of suicide, and understanding the causes of differences between different estimates of the suicide in this country.

## Materials and Methods

Iranian major scientific databases including scientific information database (SID), Magiran, Google Scholar, and PubMed were searched to find all recent 20-year original articles (April 1997- April 2016) on committed suicide. The keywords in English and Persian languages were as follow: suicide, mortality, and Iran. Those studies that focused on a specific geographic/province area, a specific method of suicide, or a specific age/sex group were excluded so that the national data from different sources could be compared with similar scales. These articles were rechecked to exclude the possible repetition of the studies. On the other hand, publications of 2 main organizations (i.e., Ministry of Health [MOH] and Iranian Forensic Medicine Organization [IFMO]) were reviewed and confronted to the published original articles to match relevant data in each institution.

***Analysis***

Descriptive data were provided to summarize the trend and the number of committed suicides. Discrepancies were highlighted and interpreted based on methodology used in each study.

## Results

The abstracts of PubMed search engine (70 results), Google Scholar (13,400 results), SID (25 results) and Magiran (43 results) were reviewed along with the publications of 2 main organizations (MOH and IFMO) responsible for reporting natural/unnatural deaths. These 2 institutions are independently dealing with health care facilities and in/out hospital unnatural deaths, respectively. Full texts were collected based on their relevance to the research criteria. In total, 135 articles were included for review. Excluding the original articles focusing on a specific method/age/sex/ or province, 9 original articles were found to describe the national attempted/committed suicide rates (Table 1) and reviewed extensively (1-9).

***Discrepancies in Suicide Rate***

According to Shirazi et al.'s study, the average rate of death by suicide was 6.7 per 100 000 with an average case fatality of 38% (7). Saberi-Zafarghandi et al. showed an increased rate of mixed suicide attempt and suicidal death rate from 8.3 to 19.4 per 100 000, followed by a subsequent decline to 16.3 per 100 000, showing a case fatality ratio of 7.3% (4). For the first time, Akbari et al. reported suicidal death rate of 6.2 per 100 000, which is much higher than IFMO (4.4 per 100 000) and MOH data (0.6 per 100,000) in 2 consecutive years (2, 6, 9). In 2009, Hajebi et al. developed a national suicide registry in Iran and recorded a suicide case fatality ratio of 3.2% (5). The last national suicide data have been recently published by Kiadaliri et al., showing an overall suicide mortality rate of around 5 per 100 000 Iranian inhabitants at the worst and last point in 2010 (1).

Kiadaliri et al. claimed that higher suicide rates in Iranian males was due to the tendency of the male attempters to hang themselves. None of the previous studies supports this claim and two-thirds of both Iranian females and males prefer suicide attempt by self-poisoning (4, 5 and 7(.

Considering all abovementioned studies, a case fatality ratio of 3.2% to 38% showed more than 11.8 times difference. The suicidal death rate (2005-2009) of 1.4-6.7 per 100 000 population also showed a difference of more than 5 times. Interestingly, while data of MOH revealed a suicide attempt rate of 16.3 per 100 000 in 2007, this rate increased to 65.8 per 100 000 in 2009, two years after establishing a national suicide registry, showing a 4-time difference in hospitalized patients. In 2008, another in-hospital study reported 166 per 100 000 registered suicide attempts (9).

***Discrepancies in Data Collection***

In Iran, reports of the health services (4, 5) and IFMO match in limited cases (1-3, 10) because both have their own limitations. Health authorities (8) never register dead bodies collected by police and those who die in hospitals days after admission may never be referred to the IFMO; and even if they are sent to IFMO, prolonged hospitalization may cover up the main cause of their death.

This difference may also originate from the fact that IFMO reports cause of death and not manner of death. That’s why they only report deaths suspected to be suicide not definite suicide deaths (10).

In a large national study, 3 methods have been considered as the most usual methods of suicide attempts: Drug overdose, narcotic overdose, and poisoning (4). However, in that study, recreational overdose-related deaths were never differentiated from self-harm. Moreover, the rate of suicide attempt may be reported higher by health authorities. On the other hand, as religious rules in Islam and cultural beliefs consider suicide a great sin, suicide reports are generally underestimated (8). Indeed, all reported suicidal attempts are only from the patients who were admitted to the hospitals.

***Gender Discrepancy***

Kiadaliri et al. presented an average male/female suicide death rate ratio of 2.34 (1). This ratio was reported to be almost 1 by MOH (4), and predominance of females was shown by Akbari et al. (7 vs. 5.9 in 100 000 inhabitants) in 2000 (6).

***Trend of Suicide***

Although the authors mentioned a higher rate of suicide commitment in Iran over the past few years, there is no information to show the trend of suicide in previous decades. WHO data from health authorities shows that suicide-related mortality rate rose from almost 0.2 in 1991 to 6.2 per 100 000 in 2003, showing a rough 31-time increase in suicide rate over 21 years (11, 12). It seems that Iran has the highest rate of suicidal death growth among Islamic countries during the recent decades. Considering young population involved, more years of lost life would be concluded in this country (6).

***Target of Preventive Strategies***

IFMO data revealed that top-list 3 provinces with highest suicidal death rates had male/female suicide ratio of 1.09 to 1.45 (10). Otherwise, lowest Human Development Indices (HDI; a main cause of suicide) belonged to three provinces with a male/female suicide ratio of 0.99 to 1.52 (1-3, 10). The current study did not present related data, but suggested that preventive resources should be targeted on males in the provinces with low socioeconomic status and low HDI. However, because the common male/female ratio of 2.34 (1) decreases in provinces with lower HDIs, suggesting a similar rate of death in males and females, we think preventive strategies should focus on the whole country with giving attention to females because they are the mainstay of the families and can change the whole lifestyle of the society.

## Discussion

Data should be interpreted according to their provider and cultural/religious beliefs, which may hide suicidal attempts and deaths. We believe the frequency of suicide deaths and attempts is higher than that reported by IFMO and MOH. Shooshtary et al. showed the lifetime prevalence of suicide attempt by interviewing 504 families. They found a lifetime incidence of 1400/100000 suicide attempts (13). Suicide attempt to committed suicide ratio differs substantially among age and gender groups as well as the selected method to commit suicide. 

**Table1:** Review of National Data on Suicide and Suicide Attempt

**Author**	**Year of estimate**	**Studied population**	**Suicide attempt rate** ^*^	**Suicide fatality rate** ^*^	**Case fatality ratio (%)**	**Source of data**	**Limitations**	**Major Findings**
Shirazi et al (7)	1981-2007	26, 768 cases of hospitalized suicide attempts from whole country	26.5	6.7	3.9	Original articles, IFMO	depends on published articles and IFMO mode of death	The mean age of suicide attempters was 25 years predominantly housekeepers and students
Akbari et al (6)	2000	10 provinces covering 16,740,637		6.2		national registry for deaths, hospitals, cemetery data, IFMO	Dead cases collected and registered by police force were not registered	Mean age of suicide was 29 years and the most common method was self-burning (2.3 per 100,000)
Moradi et al (2)	2001	Whole country		4.4		IFMO	Underestimation of hospital suicide fatality	Most of the male deaths are due to hanging and most of the female deaths are due to self-burning
Saberi-Zafarghandi et al (4)	2001-2007	Whole Country	6.4-14	0.6-1.2	7.3	MOH	The statistics are mixed regarding suicide fatality and attempts	Suicide and attempted suicides increased from 8.3 per 100, 000 in 2001 to 19.4 in 2005, and then declined to 16.3 in 2007.
Rezaeian et al (8)	2004	Whole Country		3.8 (by NOCR^*^ vs. 5.7 by MOH)		MOH and NOCR^†^	Probable underestimation of death due to cultural/religious limitations	Higher rates of suicidal deaths in the Western provinces of Iran
Sharif-Alhoseini et al (9)	2005-2008	68.9% all hospital records	166	1.7	1	Hospitals	Deaths limited to governmental hospitals at the scene of the injury or after leaving the ED were not registered.	Suicide attempts and suicide accounted for 3.96% of total injuries recorded in hospital EDs. Fatal injuries due to suicide in the EDs were 9.1% in female (36% of suicides), 5.6% in male and 3.28%, 7.56% and 2.55% in individuals aged <13, 13-65 and >65 years, respectively.
Hajebi et al (5)	2009	Most provinces covering 62,514,086 (83.6%)	65.8	2.1	3.2	MOH	Dead cases collected by police were not registered. Discrepancies are mainly due to the data collection approach	Self-poisoning was the most common method of suicide attempt (90.4%) and completed suicide (38.3%).
Kiadaliri et al (1)	2006-2010	Whole country		5^‡^		IFMO	Underestimation of hospital suicide fatality	overall gender and social disparities in the distribution of suicide mortality
Shojaei et al (3)	2010	Whole country		4.7		IFMO	Underestimation of hospital suicide fatality	Suicidal death rates are lower in comparison with most of the other countries

WHO declares that suicide may be underreported by 20% to 100%, especially in developing countries. On the other hand, it is mentioned that for every committed suicide there are at least 20 to 25 suicide attempts (14, 15). Fatality ratio of 3.2% to 7.3% reported by MOH data is approximately in accordance with other studies, indicating that 92% to 95% of the people who attempt suicide will survive (14). Gruenbaum et al. found that only 2.6% of the students reported an attempt that resulted in injury, poisoning, or overdose requiring treatment in a health care facility (16).

Previous studies revealed that the suicide rate among Islamic countries is less than the Western or even Asian countries (17). Suicide act is forbidden in Islamic cultures. On the other hand, the exact rate of attempted suicide may be underestimated because of the religious or cultural stigma and criminal offences. Twenty Islamic countries may punish suicide attempters with imprisonment, fine, or both, as well as other social deprivations (18).

Considering all restrictions, it would be logical to compare Islamic countries together. Syria, Malaysia, Kuwait, Saudi Arabia, Turkey, and Jordan have a suicidal death rate of 0.4, 0.6, 0.7, 1.3, 1.58, and 2.2 per 100,000 inhabitants among Islamic countries, respectively. According to the national suicide registry of IFMO reports, hanging, self-poisoning, and self-immolation as the first 3 methods of suicide have increased during 2006 to 2010 (19).

## Limitations

The current study lacked exploration of possible suicide risk factors such as mental disorders, economical status, drug addiction, etc., as well as method and etiologic factors. The other limitation was different methodological instruments in each study that may have led to interpretation with high probability of bias. 

## Conclusion

It is concluded that of 1400/100,000 suicide attempts reported by Shooshtary, we should expect a minimum of 36/100,000 hospital admissions for suicide attempt and a minimum 7-14/100,000 suicidal deaths. This claim was supported by Sharif-Alhoseini et al. who found 166/100,000 suicide attempts in hospitals (9). Accepting the suicidal death of 6.2/100,000 population aggregated in 2000 (6) and a 100% growth rate in MOH data and 20% in IFMO data, the suicide rates would be 7.4-12.2/100,000, and an average of 9.9/100,000 (60% growth rate), which is compatible with WHO predictions for developing countries (15).

Again, we take this opportunity to warn Iranian health policy makers that national policies for prevention of suicide are not effective enough and urgent intervention is needed, particularly for high-risk groups. ‎
